# Feature Extraction and Mapping Construction for Mobile Robot via Ultrasonic MDP and Fuzzy Model

**DOI:** 10.3390/s18113673

**Published:** 2018-10-29

**Authors:** Zhili Long, Ronghua He, Yuxiang He, Haoyao Chen, Zuohua Li

**Affiliations:** Harbin Institute of Technology Shenzhen, Shenzhen 518055, China; longzhili@hit.edu.cn (Z.L.); he.ronghua@foxmail.com (R.H.); heyuxiang1991@foxmail.com (Y.H.); hychen5@hit.edu.cn (H.C.)

**Keywords:** feature extraction, mapping construction, fuzzy classification, rotary ultrasonic array

## Abstract

This paper presents a modeling approach to feature classification and environment mapping for indoor mobile robotics via a rotary ultrasonic array and fuzzy modeling. To compensate for the distance error detected by the ultrasonic sensor, a novel feature extraction approach termed “minimum distance of point” (MDP) is proposed to determine the accurate distance and location of target objects. A fuzzy model is established to recognize and classify the features of objects such as flat surfaces, corner, and cylinder. An environmental map is constructed for automated robot navigation based on this fuzzy classification, combined with a cluster algorithm and least-squares fitting. Firstly, the platform of the rotary ultrasonic array is established by using four low-cost ultrasonic sensors and a motor. Fundamental measurements, such as the distance of objects at different rotary angles and with different object materials, are carried out. Secondly, the MDP feature extraction algorithm is proposed to extract precise object locations. Compared with the conventional range of constant distance (RCD) method, the MDP method can compensate for errors in feature location and feature matching. With the data clustering algorithm, a range of ultrasonic distances is attained and used as the input dataset. The fuzzy classification model—including rules regarding data fuzzification, reasoning, and defuzzification—is established to effectively recognize and classify the object feature types. Finally, accurate environment mapping of a service robot, based on MDP and fuzzy modeling of the measurements from the ultrasonic array, is demonstrated. Experimentally, our present approach can realize environment mapping for mobile robotics with the advantages of acceptable accuracy and low cost.

## 1. Introduction

Currently, intelligent control of indoor robots has become an essential goal. Robots require the ability to acquire environmental information and detect their own location [1]. Because most environments that robots face in real life cannot be known in advance, it is necessary to recognize the features of environmental objects and construct a surrounding map for the robot to navigate. Therefore, the technology of simultaneous localization and mapping (SLAM) has become a key development trend for indoor robots [2].

Environmental modeling can be divided into laser, visual, infrared, and ultrasound methods according to the sensor type. Laser modeling has advantages such as high accuracy, high speed, and broad detection range. However, lasers cannot accurately detect some transparent materials such as glass [3]. Thus, the laser method is mainly applied in specialized environment modeling. Visual sensors can collect huge amounts of environmental information, but the associated data processing is more time-consuming [4]. Infrared sensors are very sensitive to light in basic operation mode [5], which often results in errors in the signal to the robots. Ultrasonic sensors, which detect the environment by transmitting and receiving ultrasonic waveforms, have the advantages of sufficient detection range, accurate resolution, and stable controllability, as they are not influenced by light or the material composition of objects. Moreover, ultrasonic sensors have the competitive advantage of low cost compared with laser and visual sensors. Therefore, ultrasonic sensors are a potential solution for environment mapping for indoor mobile service robots.

The current modeling approaches to environment mapping by ultrasonic sensing include grid mapping [6,7], topological mapping [8], and geometric feature mapping [9]. Compared with other mapping approaches, feature mapping can visualize more features for secondary data processing and the mapping results are more accurate and stable. In environmental mapping construction based on feature mapping, features—including flat surfaces, corners, and cylinders—are extracted, located, and combined to generate an environmental map. The typical modeling approaches to feature extraction include the RCD algorithm [10], the arc-transversal median (ATM) method, and triangulation-based fusion (TBF). Recently, hybrid integration of feature, topological, and grid mapping has been developed [11,12]. Heinen [13] introduced a Gaussian mixture model to represent the surrounding environment and proposed a new feature-based environment mapping algorithm with the advantages of small memory usage and high processing speed, avoiding discrete errors in fast calculation. Ismail et al. [14] used the data measured by a double ultrasonic sensor and proposed a fusion algorithm for environment feature extraction in which circular-arc feature extraction was combined with Hough transform-based TBF. Lee [15] proposed a new approach to ultrasonic feature extraction modeling, which used an ultrasonic data correlation method to extract circular sets of the outer corners of objects. The integrated circle center of each set was adopted as an EKF-SLAM roadmap. However, the transmitted signals of the 12 ultrasonic sensors were sensitive to crosstalk as they used the same frequencies. Shuai et al. [16] proposed a mapping approach based on ultrasonic line segmentation. The experimental ultrasonic data were measured and then were classified by improved iterative end-point fit (IEPF). The line segments were extracted by searching for transition points, and the parameters of the line segments were estimated by Kalman filter. Finally, the line segments were integrated to generate the environmental mapping. This approach can successfully map the surrounding environment via line segments and improve the efficiency of feature extraction. However, it cannot map complex environmental features such as cylinders and corners.

In summary, although several theoretical studies have investigated ultrasonic-based environmental mapping for mobile robots, the mapping precision and computational efficiency still need to be improved. In particular, the mapping approaches for current indoor service robots focus on laser and visual sensors, which are high in cost and have some inherent negative characteristics. Meanwhile, the ultrasonic approach has many advantages such as low cost, and insensitivity to the object material and to light, making it suitable for widespread application in indoor mobile robots. Currently, the ultrasonic sensors used in mobile robots are capable of obstacle avoidance and angle error estimation [17,18], but the successful construction of mapping for an indoor robot by using ultrasonic signals has rarely been reported. Therefore, it is important to investigate new approaches with low cost and acceptable accuracy for environmental mapping.

Today, various forms of indoor mobile robots, such as automated cleaning robots, accompanying robots, and robotic waiters, are beginning to appear in ordinary life. However, these consumption-type robots face a high cost burden. In this study, we present a low-cost solution to mapping construction by using an ultrasonic array, which has a potential applicability in automated navigation for indoor mobile service robots.

The paper is organized as six sections. Section 1 presents the introduction. In Section 2, a rotating ultrasonic array platform for environmental modeling is established, and its distance data are detected and analyzed. In Section 3, conventional RCD feature extraction is analyzed and its inherent disadvantages are discussed. An MDP algorithm is proposed to optimize and improve the feature extraction. Section 4 establishes the fuzzy model to classify environmental features such as flat surfaces, corners, and cylinders. In Section 5, verification experiments of MDP and the fuzzy classification model based on the ultrasonic sensor array are described. Finally, the conclusion of this paper is presented in Section 6.

## 2. Experimental Platform Design

In order to attain the distance data of the obstacle object, the experiment platform is built, and fundamental measurements of the distance of objects at different rotary angles and with different object materials are carried out.

### 2.1. Configuration of Ultrasonic Array

**Figure 1:**
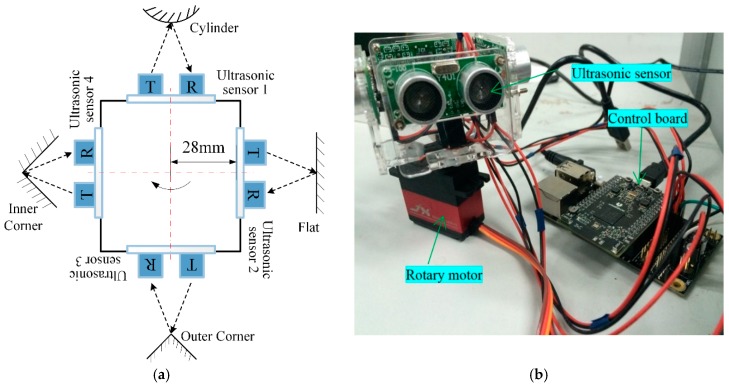
Configuration of ultrasonic array. (**a**) Schematic of ultrasonic sensor array; (**b**) experimental set-up.

The ultrasonic configuration to detect the distance between the robot and environmental objects, consisting of an ultrasonic sensor module, rotary motor, and control board, is illustrated in Figure 1. Four ultrasonic sensors are mounted on a rotary motor, in a symmetrical layout on the four sides of the motor, forming a rectangular configuration. Each ultrasonic sensor module—which includes a transmitting sensor, a receiving sensor, and the electrical control component—has the following characteristics: 300 cm maximum detection range, 3 cm dead distance, 5 mm distance resolution, and ±35° beam angle. A rotary motor with a model JX-PDI-6221MG [19] steering gear, which can rotate 90 degrees in 0.17 s and is feeding by a power supply that provides 6 V, is chosen to rotate the ultrasonic array. The functions of the control board are to control the rotary speed and direction of the motor, and transmit and receive the ultrasonic waveform by using IntoRobot Atom [20]. IntoRobot Atom is a control system with dual CPU, based on open software and hardware, offering a variety of interfaces and Wi-Fi wireless data transmission. To receive the ultrasonic signal with high precision and avoid signal crosstalk, the rotary speed of the steering gear is set to 25 ms/degree and rotates back and forth in a range of 90°. Thus, the ultrasonic array can scan the full 360° surroundings and detect the distance of environmental objects.

### 2.2. Detected Data Analysis

**Figure 2:**
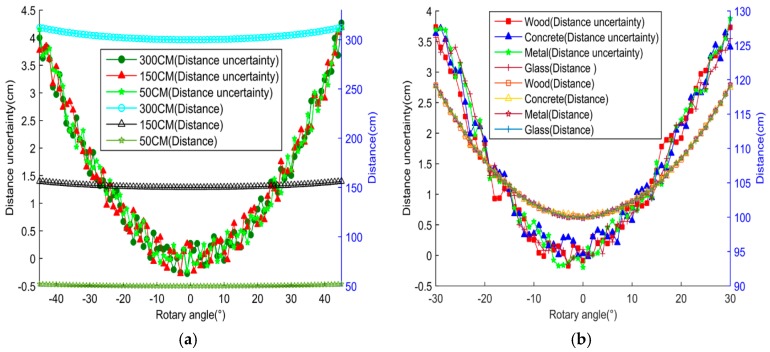
Detected data results. (**a**) Distance and its error at different angles; (**b**) distance and its error with different materials.

Figure 2a shows the detected distances, and their errors, from the array to the wall (considered as the object) when the rotary angles are ranged from −30 to +30° at 1° per step. The distance error is defined by subtracting the detected distance from the physically measured value. It is found that the rotary ultrasonic array can detect the object at distances up to 300 cm, which satisfies the detection range requirement of indoor mobile robots. The distance error increases when the rotary angle increases, but can be controlled within 20 mm in the angle range of ±30°. Figure 2b shows the distance error for different object materials—including metal, glass, concrete, and wood—at a constant physical distance of 100 cm. It can be found that the detected distance errors are consistent for these different object materials, indicating that the ultrasonic signal has adaptability to a wide range of object materials with hard surface and is suitable for object detection in indoor environments containing materials such as glass, wood, concrete, and metal.

## 3. MDP Optimization Algorithm

In order to filter or decrease the noise and disturbing data in distance measurement, some optimization algorithm is proposed.

### 3.1. Analysis of Conventional RCD

**Figure 3:**
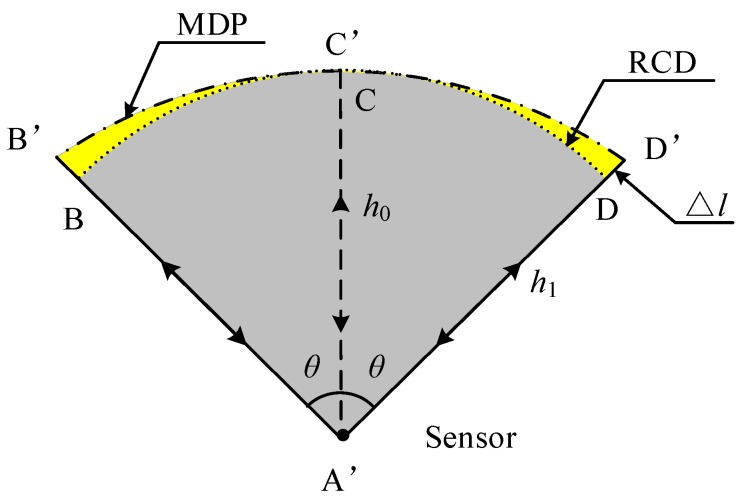
Uncertainty in the conventional RCD model.

Range of constant distance (RCD) is a conventional approach to feature recognition in robot navigation. A typical application of RCD is the distance detection of a target object by a laser sensor for automated car driving. The basic principle of distance detection of a target object is shown in Figure 3. The object locations labeled as B, C, and D are considered to be the same within the beam angle *θ*, which is symmetrical around a center line. RCD detection can achieve highly accurate measurement if using lasers, but RCD based on ultrasound would incur a distance error because of the inherent characteristics of ultrasonic signals. Distance detection by ultrasonic sensors is based on the time of flight (TOF) principle, which implies that the nearest location is detected within the beam angle. If the object is non-circular, non-equidistant, or asymmetric, the location C or *h*_0_ is detected because it is the nearest in distance, while the physical location of the expected detection is D or *h*_1_, which generates a distance bias Δ*l*. The accuracy of feature detection becomes poor if the ultrasonic sensor rotates when using the RCD method, for two reasons: (1) large error in the map outline due to the original distance error, causing the map to be narrowed or widened by RCD; and (2) estimation error when the object features are identified and classified by using the tangent circle principle. Therefore, the RCD method of ultrasonic sensing is not suitable for feature recognition and environmental modeling for mobile robots.

To compensate for the distance error Δ*l* of the conventional RCD, an approach termed minimum distance of point (MDP) is proposed. Through the geometric relationship between the object location, object type, and the location of the ultrasonic sensor, a compensated parameter Δ*l* can be calculated to attain a more accurate distance of the detected object. The compensated parameter Δ*l* is a function of the rotary angle, revolving arm length, and the object distance detected.

### 3.2. MDP Optimization for Feature Extraction

**Figure 4:**
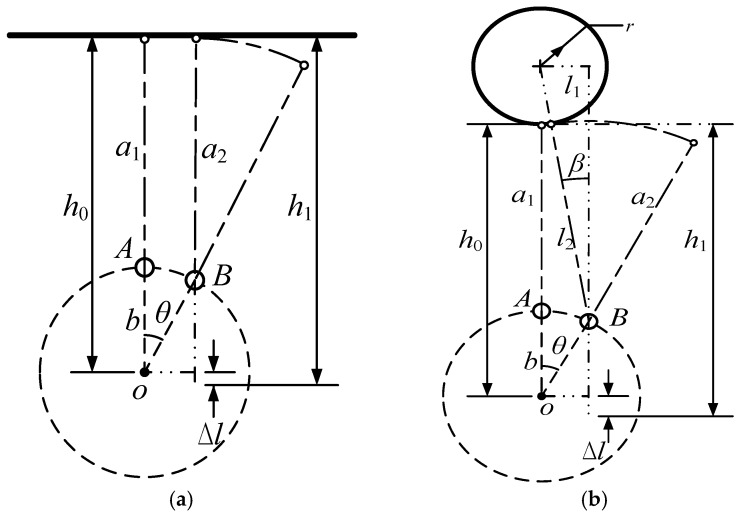
Geometric compensator model. (**a**) Flat model; (**b**) cylinder model.

To compensate for the distance error Δ*l* of the conventional RCD, an approach termed minimum distance of point (MDP) is proposed. Through the geometric relationship between the object location, object type, and the location of the ultrasonic sensor, a compensated parameter Δ*l* can be calculated to attain a more accurate distance of the detected object. The compensated parameter Δ*l* is a function of the rotary angle, revolving arm length, and the object distance detected. Figure 4 show the flat and cylinder geometric compensation model. Where, $a_{1}$ is the measurement value of point A, and $a_{2}$ is the measurement value of point B. *b* is the distance from the equivalent center of the sensor to the rotation center. $h_{0}$ is the final result of point A, and $h_{1}$ is the final result of point B. *θ* is the beam angle. *r* is the radius of cylinder.

For a flat model, the compensated distance is derived as

(1) $$\Delta l=a_{2}-a_{1}=b\left(1-\cos\theta \right)\;$$

For a cylinder model, the compensated distance is derived as

(2) $$l_{1}=b\sin\theta \;$$

(3) $$l_{2}=a_{1}+r+b(1-\cos\theta )\;$$

(4) $$\beta =arc\tan\frac{l_{1}}{l_{2}}=arc\tan\frac{b\sin\theta }{a_{1}+r+b(1-\cos\theta )}\;$$

(5) $$\begin{array}{ll} \Delta l & =\frac{l_{2}}{\cos\beta }-l_{2}+b(1-\cos\theta )\\ & =\frac{a_{1}+r+b(1-\cos\theta )}{\cos\left(arc\tan\frac{b\sin\theta }{a_{1}+r+b(1-\cos\theta )}\right)}-a_{1}-r \end{array}$$

Compared with the conventional RCD, the MDP algorithm not only can obtain a more accurate object distance, but also can extract the other features of the target object for environment mapping. In MDP, the first step to recognize the object features is to attain, from within the original distance dataset, the minimum-distance point that can express the object location. If the minimum distance were directly chosen from the original data, a random error would arise. To improve the accuracy and stability of the minimum-distance point, the least-squares polynomial fitting method is adopted in our study. The *m*-degree polynomial fitting expression can be given as

(6) $$y=a_{0}+a_{1}x+a_{2}x^{2}+\cdots +a_{m}x^{m}\;$$

where *y* are the detected distance values with compensated parameter Δ*l* at each rotary angle *x*. *m* is the index of the detected data. *a*_0_~*a_m_* is the optimal parameter in the fitting model. The quadratic sum of the error *e* is calculated according to the least-squares method

(7) $$e^{2}={\sum_{i=1}^{n}\left[y_{i}-\left(a_{0}+a_{1}x_{i}+\cdots +a_{m}x_{i}{}^{m}\right)\right]}^{2}\;$$

Then, partial differentiation with respect to *α*_0_~*α_m_* yields the optimal estimation, as

(8) $$\{\begin{array}{c} -2\sum_{i=1}^{n}\left[y_{i}-\left(a_{0}+a_{1}x_{i}+\cdots +a_{m}x_{i}{}^{m}\right)\right]=0\\ -2\sum_{i=1}^{n}\left[y_{i}-\left(a_{0}+a_{1}x_{i}+\cdots +a_{m}x_{i}{}^{m}\right)\right]x_{i}=0\\ \vdots \\ -2\sum_{i=1}^{n}\left[y_{i}-\left(a_{0}+a_{1}x_{i}+\cdots +a_{m}x_{i}{}^{m}\right)\right]x_{i}{}^{m}=0 \end{array}\;$$

In matrix form, the above can be expressed as

(9) $$\left[\begin{array}{cccc} n & \sum_{i=1}^{n}x_{i} & \cdots & \sum_{i=1}^{n}x_{i}^{m}\\ \sum_{i=1}^{n}x_{i} & \sum_{i=1}^{n}x_{i}^{2} & \cdots & \sum_{i=1}^{n}x_{i}^{m+1}\\ \vdots & \vdots & \ddots & \vdots \\ \sum_{i=1}^{n}x_{i}^{m} & \sum_{i=1}^{n}x_{i}^{m+1} & \cdots & \sum_{i=1}^{n}x_{i}^{2m} \end{array}\right]\left[\begin{array}{c} a_{0}\\ a_{1}\\ \vdots \\ a_{m} \end{array}\right]=\left[\begin{array}{c} \sum_{i=1}^{n}y_{i}\\ \sum_{i=1}^{n}x_{i}y_{i}\\ \vdots \\ \sum_{i=1}^{n}x_{i}^{m}y_{i} \end{array}\right]\;$$

The above Vandermonde matrix is simplified to attain

(10) $$\left[\begin{array}{cccc} 1 & x_{1} & \cdots & x_{1}^{m}\\ 1 & x_{2} & \cdots & x_{2}^{m}\\ \vdots & \vdots & \ddots & \vdots \\ 1 & x_{n} & \cdots & x_{n}^{m} \end{array}\right]\left[\begin{array}{c} a_{0}\\ a_{1}\\ \vdots \\ a_{m} \end{array}\right]=\left[\begin{array}{c} y_{1}\\ y_{2}\\ \vdots \\ y_{n} \end{array}\right]\;$$

**Figure 5:**
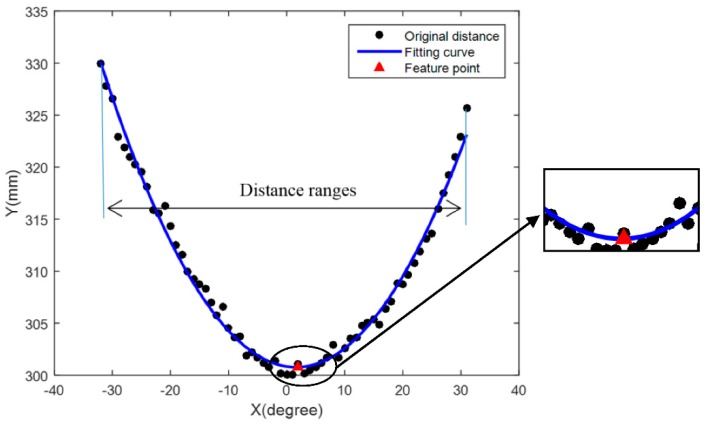
Determination of object location by MDP fitting.

Equation (10) can be expressed as X×A=Y, so the coefficient matrix with the optimal parameters is attained as

(11) $$A={\left(X^{'}\times X\right)}^{-1}\times X^{'}\times Y\;$$

where matrix *X* is the dataset of rotary angles, and matrix *Y* is the dataset of corresponding distances with compensated parameters Δ*l*. Therefore, when the rotary angles and corresponding distances detected are substituted into the above expression, the fitting expression can be established and the location point of the target object can be determined. Figure 5 shows the result of polynomial fitting to one of the original datasets in our study. Thus, the object location and the ultrasonic distance data can be determined and regarded as the input dataset to further classify the object feature types such as flat surfaces, corners, and cylinders.

## 4. Feature Classification Based on Fuzzy Model

**Figure 6:**
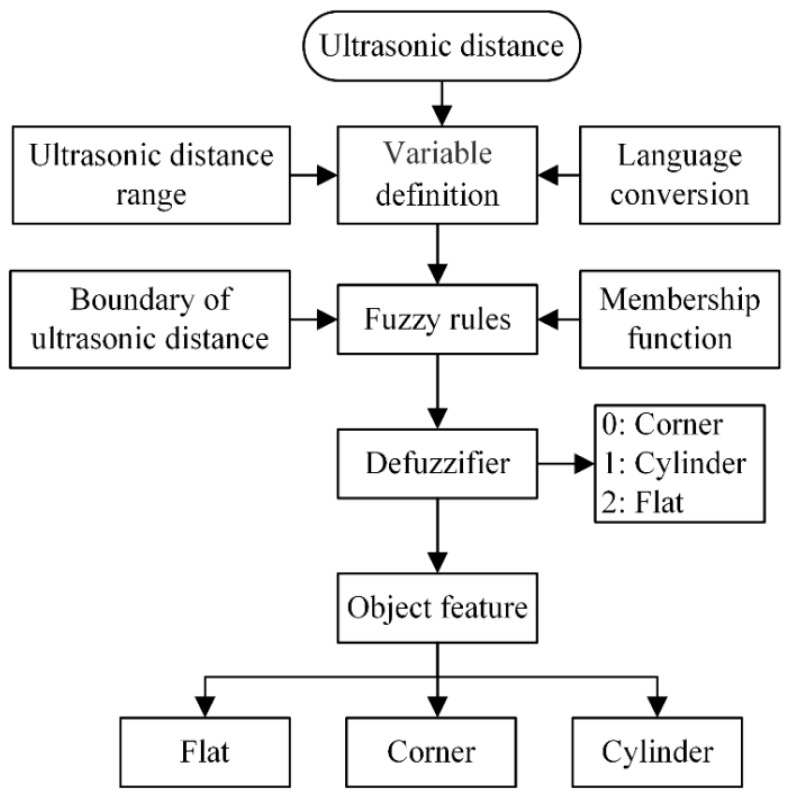
Procedure of fuzzy classification model.

Fuzzy theory, which was introduced by Zadeh [21], is a mature solution in motion control, signal processing, and artificial intelligence. The principle of fuzzy theory is to attain an analytical result by applying expert knowledge and practical experience in the form of reasoning rules. Although fuzzy theory is a conventional algorithm, it has many advantages, such as no requirement for a physical expression, less computational time, and strong robustness [22,23,24]. Generally, building a fuzzy controller consists of the following steps: variable definition, fuzzification, implementation of fuzzy rules, and defuzzification. In this study, a fuzzy model is proposed to recognize and classify object feature types such as corners, flat surfaces, and cylinders. Figure 6 shows the procedure of fuzzy modeling for feature classification. Firstly, the object features are scanned and the distance dataset is attained by the ultrasonic array. The object location is determined by MDP compensation. Also, the ranges of the distance dataset are regarded as the input variables of the fuzzy model. Secondly, the distance data are treated by fuzzification and the reasoning rules relating to boundary state are established. Finally, the types of object feature can be recognized and classified as the outputs of the fuzzy model.

### 4.1. Variable Definition and Language Conversion

#### 4.1.1. Ultrasonic Distance Range

As implicit in the data measured by the ultrasonic sensor array, the range of the distance dataset has a relationship with the objects’ geometric features. In our study, the flat surfaces have the maximum data range, corners the smallest, and the range for cylindrical objects is medium. Thus, the language conversion and data fuzzification can be expressed as in Table 1.

#### 4.1.2. Boundary Definition

The boundary of the fuzzy model can be defined by the covering state (i.e., covered or uncovered) of the distance detected. The range of the distance dataset is reduced if some of the distance detected is covered. Table 2 shows the boundary states of the distance detected in our study, including the non-covering, left-covering, right-covering, and front-covering states, with corresponding setting values of 0, 1, 2 and 3, respectively.

#### 4.1.3. Output Feature Types

As shown in Table 3, the classification of features as corner, cylinder, and flat surface, which are the output of the fuzzy model, are defined as 0, 1, and 2, respectively.

### 4.2. Data Fuzzification

Data fuzzification is a process to convert the input and output variables into suitable linguistic values. In the fuzzy model, it is essential to fuzzify the ultrasonic distance ranges. Because the detected distance dataset may be fluctuant, the upper limit *L*_max_ of the distance dataset is defined as the average of the maximum values *L*_max 1_, *L*_max 2_ of two repeated scans, as

(12) $$L_{\max}=\frac{L_{\max1}+L_{\max2}}{2}\;$$

Based on the results of our practical testing, the maximum membership degrees of the big, medium, and small states, which can be expressed by *L*_b_, *L*_m_, and *L*_s_, are defined as

(13) $$\left\{ \begin{array}{l} L_{b}=L_{\max}\times 0.9\\ L_{m}=L_{\max}\times 0.55\\ L_{s}=L_{\max}\times 0.15 \end{array}\right.\;$$

The membership function of ultrasonic distance range is attained

(14) $$\mu_{0}=\left\{ \begin{array}{ll} 1, & 0\leq u\leq L_{s}\\ \frac{-u}{L_{l}-L_{s}}+\frac{-L_{l}}{L_{s}-L_{l}}, & L_{s}<u \end{array}\right.\;$$

(15) $$\mu_{1}=\left\{ \begin{array}{l} \frac{u}{L_{m}-L_{s}}+\frac{L_{l}}{L_{s}-L_{m}},L_{s}<u<L_{m}\\ \frac{-u}{L_{b}-L_{m}}+\frac{-L_{b}}{L_{m}-L_{b}},L_{m}\leq u<L_{b} \end{array}\right.\;$$

(16) $$\mu_{2}=\left\{ \begin{array}{ll} \frac{u}{L_{l}-L_{s}}+\frac{L_{s}}{L_{s}-L_{l}}, & L_{m}<u<L_{l}\\ 1, & L_{l}\leq u \end{array}\right.\;$$

(17) $$\left\{ \begin{array}{l} L_{1}=\frac{L_{b}L_{s}-L_{s}^{2}}{-2L_{s}+L_{b}+L_{m}}\\ L_{2}=\frac{L_{b}^{2}-L_{s}L_{m}}{2L_{b}-L_{s}-L_{m}} \end{array}\right.\;$$

**Figure 7:**
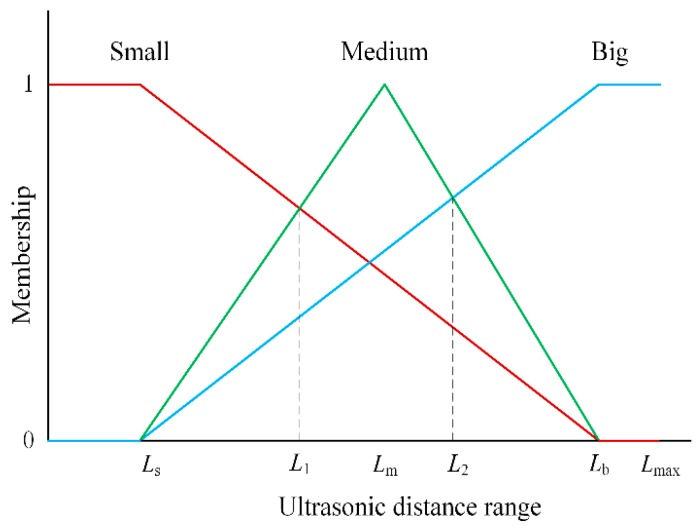
Membership of ultrasound distance range.

All of the input variables are normalized into [0, 1]. The linguistic values of the inputs and outputs are quantified using the membership functions. Thus, the membership curve can be established as in Figure 7.

### 4.3. Fuzzy Rules and Defuzzification

Fuzzy rules are used within fuzzy logic systems to deduce an output based on input variables, and defuzzification is the process of producing a fuzzy set into a crisp number.

#### 4.3.1. Fuzzy Rules

The boundaries of ultrasonic distance, and their state (i.e., covered or not), have a significant influence on the ultrasonic distance range. Therefore, the effect of boundary state on ultrasonic distance range must be considered in the reasoning rules of the fuzzy model. It is necessary to redefine the membership of ultrasonic distance range at different boundary states, as shown in Table 4.

In Table 4, when the ultrasonic distance range is 2 with the big setting, the memberships of ultrasonic distance keep constant when the boundary is 0, i.e., non-covering. They also keep constant even when the boundary is 1, 2 and 3, because in that case the ultrasonic distance range has a strong stable ability to avoid being disturbed by the covering. When the ultrasonic distance range is 0 with the small setting, the membership is decreased by 0.25 if the boundary is 1 (left covering) or 2 (right covering). The membership is decreased by 0.30 if the boundary is 3 (front covering). Likewise, the membership redefinition of distance range 1 in different covering states can be deduced by similar rules.

#### 4.3.2. Defuzzification

When the maximum membership degree is *μ*_0_, the output of the fuzzy model is defined as 0 for a corner feature. Similarly, the outputs of the fuzzy classification model are 1 for a cylindrical feature, and 2 for a flat surface, when the maximum membership degree is *μ*_1_ and *μ*_2_, respectively.

## 5. Experiment and Verification

**Figure 8:**
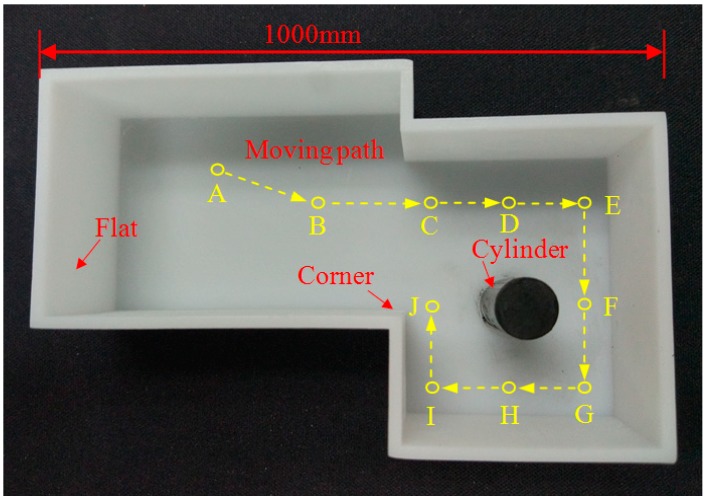
Environment for feature classification and mapping.

**Figure 9:**
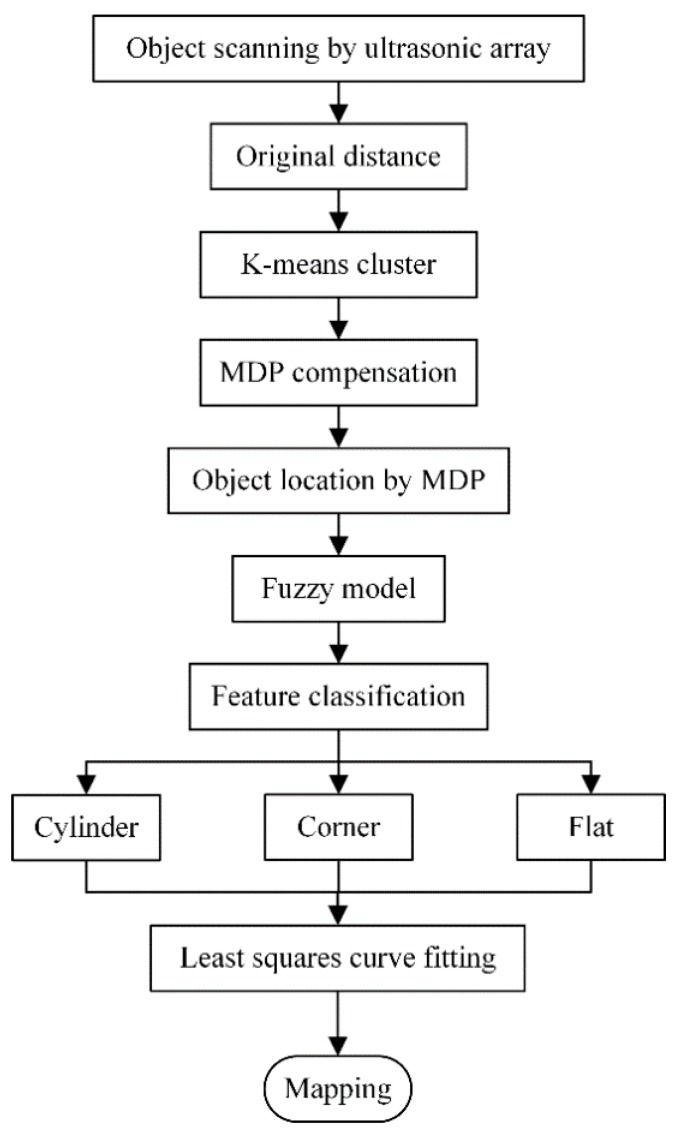
Flowchart of feature extraction, classification, and mapping.

To verify the feasibility of the MDP algorithm and fuzzy model applied in feature classification for mobile robotics, experimental testing and verification analysis are carried out. The environment for classification and mapping is designed as in Figure 8, and includes flat surfaces, corner, and cylinder features. A mobile robot mounted with the ultrasonic sensor array travels by a defined trajectory from A, B, C, …, to J. In the experiment, the ultrasonic distance data are detected and preprocessed by some filter method such as K-means cluster [25]. The location and the distance range of the ultrasonic dataset are extracted by the MDP algorithm. Then, the fuzzy model is utilized to classify the features of flat surface, cylinder, and corner. Finally, line fitting, least-squares curve fitting, and integrated combined methods are adopted to generate and construct the environmental map. The flowchart of the feature extraction, feature classification, and mapping is shown in Figure 9.

### 5.1. Distance Data Detection and Preprocessing

**Figure 10:**
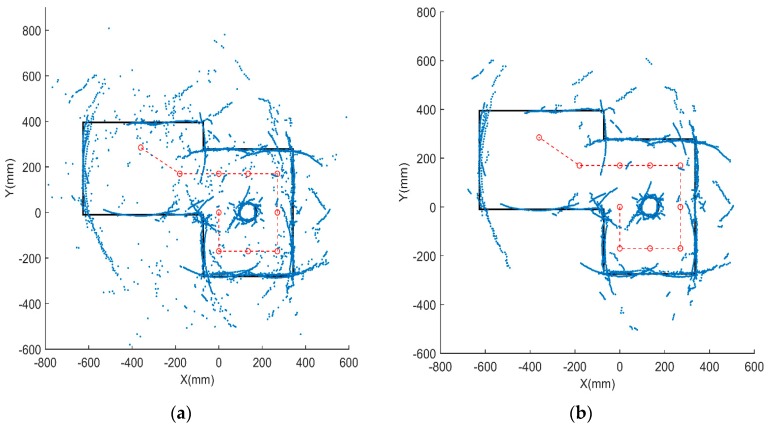
Preprocessing of original data. (**a**) Original distance dataset; (**b**) distance dataset after preprocessing.

In our experiment, 10 groups of ultrasonic distance data are measured and the original data are visualized by MATLAB, as shown in Figure 10a. It is found that the original distance dataset includes some noise and regions of discrete distribution. To reduce the noisy data, a K-means filter is utilized to cluster the original data, and the result is shown in Figure 10b. It can be seen that the noisy and discrete data are cleared and the ultrasonic distance data representing the line and arc features are displayed in outline.

### 5.2. Feature Extraction and Classification

**Figure 11:**
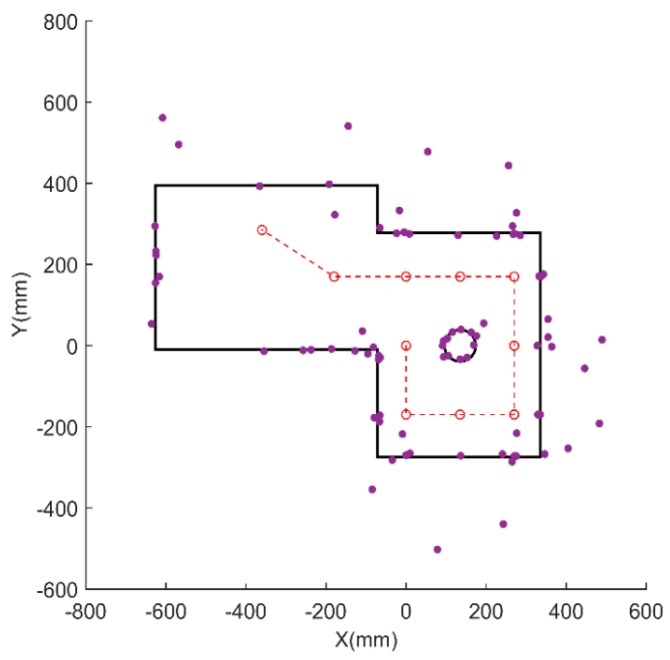
Object location extraction by MDP.

Having attained the ultrasonic distance and its range by data preprocessing, the distance data are compensated further by the MDP method to achieve more accuracy. Then, the MDP algorithm with least-squares polynomial fitting is applied to extract the minimum distance of the distance dataset, allowing the object location to be determined. The result is shown in Figure 11. The preprocessed distance data are clustered again by the differential distance clustering method. It is also confirmed that the ranges of the flat surfaces, cylinder, and corner are big, medium, and small, respectively.

**Figure 12:**
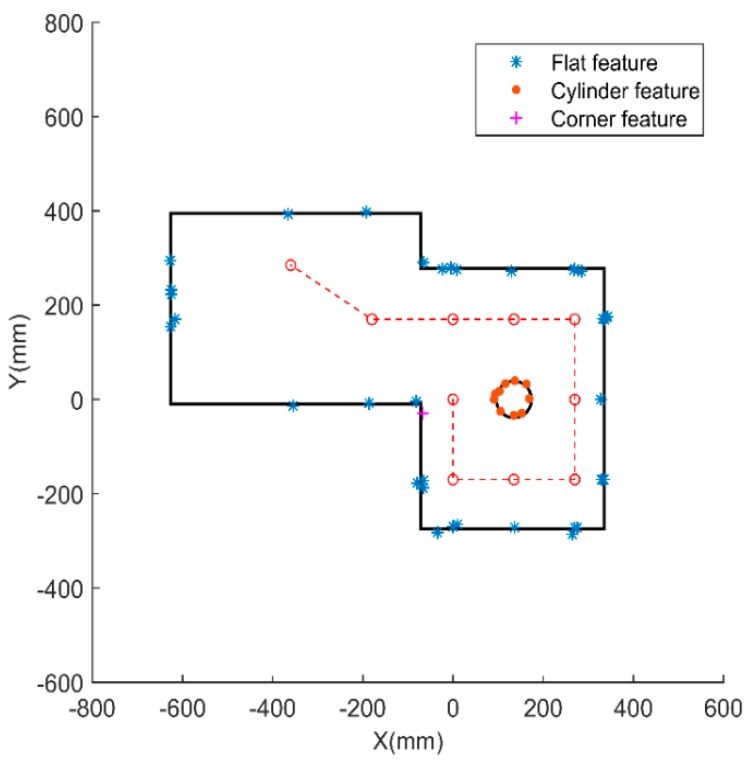
Feature classification by fuzzy model.

The clustered ultrasonic distance range and the boundary state are input into the fuzzy classification model. After the data fuzzification, reasoning, and defuzzification, the feature types, that is, the flat surfaces, cylinders, and corners, are output as the classification result. In our experiment, eight flat features, one cylindrical feature, and one corner feature are attained by fuzzy classification. The classification results are shown in Figure 12.

### 5.3. Mapping Construction

**Figure 13:**
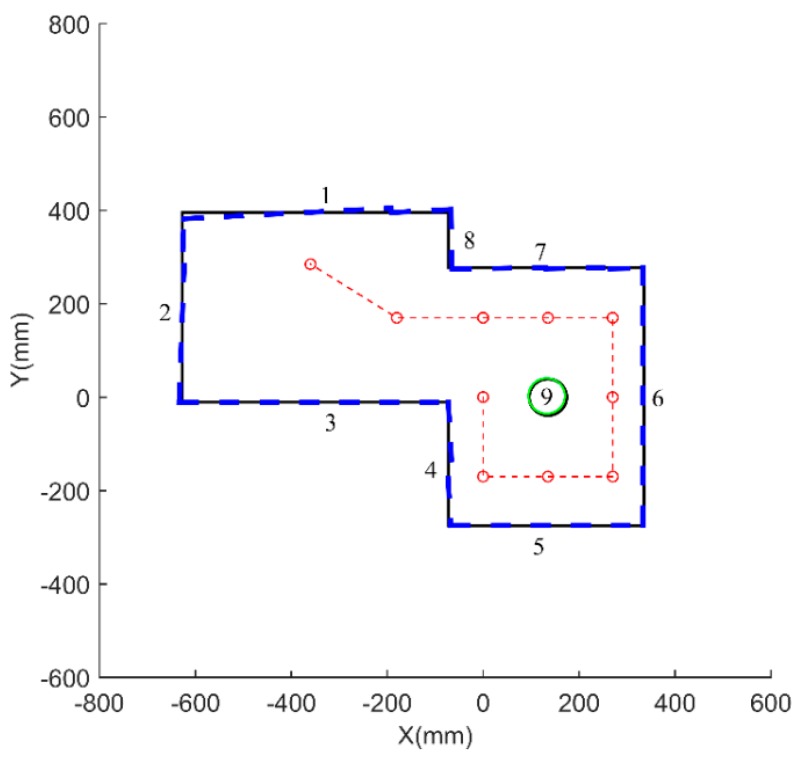
Mapping construction result.

The line fitting method is used to connect and form straight lines, an arc, and a corner. In our study, eight straight lines representing the flat features are generated, and the intersections between the straight lines are calculated. Least-squares curve fitting is performed to generate an arc, which represents the cylinder feature. The corner feature is generated by the intersection of two lines. Finally, a comprehensive environment map is constructed successfully, as shown in Figure 13.

To evaluate the mapping accuracy, the map constructed by MDP and the fuzzy model is compared with the physically measured map. The comparison results are summarized in Table 5. It is shown that, in our designed environment and the setting parameters in fuzzy model, the flat surface with index 1 has the highest distance deviation of 12.8 mm, and its angle deviation is 3°. The flat surface with index 5 has the lowest distance deviation of 0.5 mm and its angle deviation is 0°. In this example studied with basic geometry, it is verified that the MDP and fuzzy model can realize map construction with high accuracy and multiple features by using the rotary ultrasonic sensor array.

## 6. Conclusions

In this paper, an ultrasonic sensor array with MDP and fuzzy modeling is developed as a feasible, low-cost approach for environment modeling of an indoor mobile robot. A configuration with a rotary ultrasonic sensor array is design to detect the distance of objects in a 360° scope. The MDP algorithm and fuzzy model are proposed to extract and classify environmental features such as flat surfaces, cylinder, and corner. The conclusions are summarized as follows.

(1) The MDP feature extraction algorithm is proposed and the least-squares polynomial curve fitting is applied to extract the minimum-distance point of the ultrasonic distance dataset. This MDP method can compensate for the feature location error in the conventional RCD algorithm.
(2) A feature classification model based on fuzzy theory is established. The ultrasonic distance range and distance state detected by the ultrasonic sensor are regarded as the input of the fuzzy model. The rules of data fuzzification, reasoning, and defuzzification are defined according to practical testing experience. With the fuzzy model, the classification results—including flat surfaces, a corner, and a cylindrical feature—can be attained.
(3) The object feature extraction and the mapping construction by using MDP and the fuzzy model are successfully verified by experiment. A fully accomplished case of mapping with MDP and the fuzzy model is demonstrated in our study using basic regular geometry. Compared with the physical map, the distance error constructed by the rotary ultrasonic array is 1.2 cm and the angle error is 3°, representing high accuracy of environmental mapping for indoor mobile robotics.

## Figures and Tables

**Table 1:** Language conversion of ultrasonic distance range

**State**	Small	Medium	Big
**Value**	0	1	2

**Table 2:** Boundary definition

**State**	Non-Covering	Left Covering	Right Covering	Front Covering
**Value**	0	1	2	3

**Table 3:** Language conversion of feature types

**State**	Corner	Cylinder	Flat
**Value**	0	1	2

**Table 4:** Membership redefinition of ultrasonic distance range

Membership		Boundary State			
		0	1	2	3
**Ultrasonic distance range**	0	*μ*_0_	*μ*_0_ − 0.25	*μ*_0_ − 0.25	*μ*_0_ − 0.3
		*μ*_1_	*μ*_1_ + 0.25	*μ*_1_ + 0.25	*μ*_1_
		*μ*_2_	*μ*_2_	*μ*_2_	*μ*_2_ + 0.3
	1	*μ*_0_	*μ*_0_	*μ*_0_	*μ*_0_
		*μ*_1_	*μ*_1_ − 0.2	*μ*_1_ − 0.2	*μ*_1_ − 0.3
		*μ*_2_	*μ*_2_ + 0.2	*μ*_2_ + 0.2	*μ*_2_ + 0.3
	2	*μ*_0_	*μ*_0_	*μ*_0_	*μ*_0_
		*μ*_1_	*μ*_1_	*μ*_1_	*μ*_1_
		*μ*_2_	*μ*_2_	*μ*_2_	*μ*_2_

**Table 5:** Accuracy of the mapping result

Index	Distance Uncertainty (mm)	Angle Uncertainty (°)
1	12.8	3
2	6.7	2
3	1.0	0
4	7.8	3
5	0.5	0
6	1.8	0
7	3.8	1
8	7.0	1
